# Bioinspired and Photo-Clickable Thiol-Ene Bioinks for the Extrusion Bioprinting of Mechanically Tunable 3D Skin Models

**DOI:** 10.3390/biomimetics9040228

**Published:** 2024-04-10

**Authors:** Luís B. Bebiano, Rafaela Presa, Francisca Vieira, Bianca N. Lourenço, Rúben F. Pereira

**Affiliations:** 1 i3S—Instituto de Investigação e Inovação em Saúde, Universidade do Porto, Rua Alfredo Allen, 208, 4200-135 Porto, Portugal; 2 INEB—Instituto de Engenharia Biomédica, Universidade do Porto, Rua Alfredo Allen, 208, 4200-135 Porto, Portugal; 3 ICBAS—Instituto de Ciências Biomédicas Abel Salazar, Universidade do Porto, Rua Jorge de Viterbo Ferreira, 228, 4050-313 Porto, Portugal

**Keywords:** bioink, skin bioprinting, click chemistry, in vitro model, extrusion bioprinting, mechanical cues, dermis, epidermis, pectin, hydrogels

## Abstract

Bioinks play a fundamental role in skin bioprinting, dictating the printing fidelity, cell response, and function of bioprinted 3D constructs. However, the range of bioinks that support skin cells’ function and aid in the bioprinting of 3D skin equivalents with tailorable properties and customized shapes is still limited. In this study, we describe a bioinspired design strategy for bioengineering double crosslinked pectin-based bioinks that recapitulate the mechanical properties and the presentation of cell-adhesive ligands and protease-sensitive domains of the dermal extracellular matrix, supporting the bioprinting of bilayer 3D skin models. Methacrylate-modified pectin was used as a base biomaterial enabling hydrogel formation via either chain-growth or step-growth photopolymerization and providing independent control over bioink rheology, as well as the mechanical and biochemical cues of cell environment. By tuning the concentrations of crosslinker and polymer in bioink formulation, dermal constructs were bioprinted with a physiologically relevant range of stiffnesses that resulted in strikingly site-specific differences in the morphology and spreading of dermal fibroblasts. We also demonstrated that the developed thiol-ene photo-clickable bioinks allow for the bioprinting of skin models of varying shapes that support dermis and epidermis reconstruction. Overall, the engineered bioinks expand the range of printable biomaterials for the extrusion bioprinting of 3D cell-laden hydrogels and provide a versatile platform to study the impact of material cues on cell fate, offering potential for in vitro skin modeling.

## 1. Introduction

Bioprinting is one strategy used in the field of biofabrication to create 3D constructs with biological function for tissue engineering and regenerative medicine [1]. In the context of skin tissue engineering, the ability of bioprinting to control the positioning of biomaterials, cells, and bioactive agents enables the automated fabrication of engineered biological constructs that recapitulate essential properties and functions of human skin. Such constructs are used as implants to restore or replace damaged skin and as in vitro models of healthy or diseased skin for several purposes, ranging from fundamental studies to drug discovery and screening [2,3,4,5,6].

Some of the bioprinting technologies explored to create skin constructs include light-assisted, inkjet-, and extrusion-based bioprinting. Light-assisted bioprinting uses light to promote the sol–gel transition of a low viscosity and photocrosslinkable bioink into a 3D construct. It is characterized by a high resolution and requires photocrosslinkable biomaterials with suitable processability and cell compatibility [7]. Inkjet-based bioprinting comprises the deposition of bioinks via a droplet-wise dispensing mechanism through piezoelectric or thermal effects. Despite enabling cell deposition with high resolution, inkjet-based bioprinting is limited to bioinks with low cell density and viscosity, to preclude clogging issues [8]. As a result, it has been widely explored for epidermis reconstruction via the controlled deposition of epidermal cells [9]. Extrusion-based bioprinting is the most common technology for creating 3D skin constructs, due to its ability to print bioinks with a broad range of rheological properties into centimeter-sized 3D constructs with reasonable resolution and controllable mechanical characteristics. The traditional approach relies on the direct deposition of self-supporting bioinks into personalized implants and skin models. Despite this being technologically feasible using common bioprinters, it requires the fine tuning of bioink rheology to enable the deposition of continuous and mechanically robust filaments that hold their shape during bioprinting, without impairing long-term cell viability and function [10]. In addition, to assist the bioprinting of biologically functional skin constructs, it is essential that bioinks provide the embedded cells with tissue-like mechanical properties and biochemical cues to promote spatial cell organization and stimulate neo-tissue formation with mechanical integrity, as seen in native skin.

Several biomaterials have been proposed to design bioinks for the extrusion bioprinting of skin constructs, including collagen, gelatin, fibrin, pectin, hyaluronic acid, and decellularized extracellular matrix (ECM) [11,12,13,14,15,16]. Protein-derived bioinks, such as those based on collagen, remain the gold standard, as collagen is the prime component of skin ECM and inherently supports cell attachment and remodeling [9,17]. However, collagen-based constructs are often characterized by weak mechanical properties and extensive cell-mediated mechanical contraction, which results in uncontrolled dimensional changes that might alter the predictive value and biological function of bioprinted constructs [18,19]. A common strategy to address these issues relies on the design of multimaterial bioinks by merging the biological cues of protein-derived biomaterials with the specific characteristics of polysaccharides, including their tunable rheology and mechanical properties [4]. Polysaccharides, like alginate and hyaluronic acid, are typically used as rheology modifiers to improve the printability of protein-based bioinks by tuning their crosslinking degree, molecular weight, or concentration [20]. Alternatively, these biomaterials can integrate the bioprinted construct, imparting mechanical properties [21]. Despite having been widely explored, this strategy increases the complexity in bioink design, often requires temperature control during bioprinting to avoid premature gelation, and, in some cases, severely limits the ability to precisely control the presentation of defined biochemical and biomechanical cues to the cells.

This study reports the rational design and fabrication of ECM-mimetic, thiol-ene photo-clickable bioinks with defined rheology, controlled biochemical cues, and tunable mechanical properties that support the bioprinting of dermal and dermo–epidermal skin models. The strategy synergizes the reversible nature of ionic gelation with the fast kinetics and regioselectivity of thiol-ene click chemistry to independently control the printability, adhesive ligand presentation, and protease-dependent degradation of bioinks. Whereas previous studies have focused on multimaterial bioinks for skin bioprinting [4,12,15], we hypothesized that by combining pectin as an ionically crosslinkable polymer, complementary crosslinking reactions, and relevant peptide sequences that recreate fundamental dermal ECM properties, it is possible to create single biomaterial bioinks with tunable properties that enable the presentation of controlled mechanical cues to skin cells. To do so, we developed a series of pectin-based bioinks and demonstrated that bioprinted cell-laden hydrogels can be rapidly photocrosslinked via either chain- or step-growth mechanisms by adding a peptide crosslinker to the bioink formulation. Then, we showed that the viscoelasticity of bioprinted hydrogels can be easily tuned, eliciting significant differences in cell response in 3D and promoting the de novo deposition of dermal ECM. Lastly, bilayer skin models of different shapes were bioprinted as a proof-of-concept demonstration of the bioink ability in supporting in vitro skin reconstruction.

## 2. Materials and Methods

### 2.1. Polymer Synthesis

Pectin methacrylate (PECMA) was synthetized according to an established protocol with minor modifications [5]. Briefly, purified low-methoxyl citrus pectin with a galacturonic acid unit content of 86% and a degree of methylation of 37% (Classic CU701, Herbstreith & Fox, Neuenbürg, Germany) was reacted with methacrylic anhydride (MA, Sigma-Aldrich, St. Louis, MO, USA) in phosphate-buffered saline (PBS, pH 7.4) for 24 h with periodic pH adjustment to 8.0 using 5 M sodium hydroxide (NaOH). Modified polymer was precipitated in cold acetone and was centrifuged. Then, the polymer was resuspended in ultrapure water and dialyzed (MWCO 3500, Spectra/Por^®^, SpectrumLabs, Auckland, New Zealand) for 5 days. Finally, the pH of the polymer solution was adjusted to 7.00 using 0.1 M NaOH, filtered (0.20 μm), and the PECMA polymer was recovered using lyophilization. The extent of methacrylate substitution was determined using ^1^H NMR, recorded with a 400 MHz spectrometer AVANCE III (Bruker, Billerica, MA, USA) [5].

### 2.2. Cell Culture

Human neonatal dermal fibroblasts (hNDFs) isolated from human neonatal foreskin samples (Coriell Institute for Medical Research) and the cell line keratinocytes (HaCaT) were cultured in DMEM (31966, Gibco, Billings, MT, USA) supplemented with 10% fetal bovine serum (FBS, 10270, Gibco), antibiotics (100 U/mL penicillin, 100 μg/mL streptomycin, Sigma-Aldrich), and amphotericin B (2.5 mg/L, Sigma-Aldrich). Cells were cultured in 5% CO_2_ at 37 °C in tissue culture polystyrene flasks. Cells were trypsinized using a 0.05 wt% trypsin/ethylenediamine tetraacetic acid (EDTA) solution before reaching confluence (70–80%) and centrifuged at 245 rcf for 5 min.

### 2.3. Bioink Preparation and Photocrosslinking

Bioinks were composed of PECMA (1.5 or 2 wt%), 2-hydroxy-4-(2-hydroxyethoxy)-2-methylpropiophenone as a photoinitiator (IR2959, 0.05 wt%, Sigma-Aldrich), peptides (MMP-degradable peptide crosslinker: CGPQGIWGQC (0.5 or 4 mM) and cell-adhesive peptide ligand CGGGGRGDSP (2 mM), GenScript Biotech, Netherlands), and hNDFs. Upon polymer dissolution in IR2959 dissolved in 0.9% sodium chloride (NaCl), the required peptides were gently mixed and calcium chloride dihydrate (CaCl_2_·2H_2_O, 6 mM) was added dropwise under agitation, followed by vigorous mixing to promote uniform Ca^2+^ distribution and ionic crosslinking. After 1 h, 1.0 × 10^7^ cells/mL were carefully mixed within the pre-crosslinked PECMA at 20 °C. For photocrosslinking, bioinks and cell-free pre-crosslinked gels (inks) were exposed to UV light (7 mW/cm^2^, 365 nm, BlueWave 200, Dymax, Torrington, CT, USA) for the desired time periods. For sterile culture studies, all solutions were filter-sterilized using a 0.22 μm filter.

### 2.4. Rheological Measurements and Mechanical Testing

Rheological and viscoelastic properties of acellular calcium-crosslinked and double crosslinked hydrogels were determined using a Kinexus Pro rheometer (Malvern, Malvern, UK). Rotational shear-viscosity measurements were carried out at 25 °C using a shear ramp test (1–1000 s^−1^, 2 min) after applying one loading cycle with 2 min intervals, before acquiring the rheological data. The yield stress, which is the minimum shear stress required to initiate flow, was analyzed by applying a shear stress ramp (1–100 Pa, 2 min) with plate–plate geometry (0.5 mm) at 25 °C. Viscoelastic properties of double crosslinked hydrogels equilibrated in culture medium at 5% CO_2_ and 37 °C for 24 h were determined by testing 4 mm diameter samples in a humidified environment at 37 °C. Samples were compressed at 20% of their initial height and strain amplitude sweeps were conducted from 0.1 to 100% at 0.1 Hz, while frequency sweeps were carried out from 0.01 to 10 Hz at 1% strain (within the linear viscoelastic region).

### 2.5. Bioprinting of 3D Skin Equivalents

Bioprinting was performed using the bioprinter Regemat 3D V1 (Regemat 3D, Granada, Spain). To create the dermal layer, the bioink composed of PECMA (1.5 or 2 wt%), IR2959 (0.05 wt%), CaCl_2_·2H_2_O (6 mM), CGGGGRGDSP peptide (2 mM), CGPQGIWGQC peptide (0.5 or 4 mM), and hNDFs (1.0 × 10^7^ cells/mL) was bioprinted under the following conditions: 4 mm/s printing speed, 25 G tapered nozzle (Nordson EFD, Westlake, OH, USA), and 4 layers (1 mm thick). Then, the constructs were photocrosslinked for 40 s and incubated in culture medium at 5% CO_2_ at 37 °C. For epidermis reconstruction, cell line keratinocytes (HaCaT) were seeded onto the dermal layer (5.0 × 10^5^ cells/cm^2^), left to attach for 2 h, and cultured submerged for 7 days. Then, the constructs were cultured at the air–liquid interface (ALI culture) for 14 days. Culture medium was changed every two days. The dermis was bioprinted either directly onto a 24-well plate transwell insert (Corning, Corning, NY, USA) by depositing 4 layers or by the free form bioprinting of solid square constructs (6 × 6 × 1 mm^3^ or 8 × 8 × 1 mm^3^).

### 2.6. Characterization of Cell Response in 3D Hydrogels

The metabolic activity of embedded cells was assessed using the resazurin assay through incubation of cell-laden gels in DMEM medium containing 20% *v*/*v* resazurin sodium salt (Sigma-Aldrich) for 2 h at 37 °C. Samples were measured using a microplate reader (Synergy MX, Biotek, Winooski, VT, USA) at 530 nm (excitation) and 590 nm (emission). To evaluate cell morphology and fibronectin deposition in the dermis, cell-laden hydrogels were fixed in 4% *v/v* paraformaldehyde (PFA, Electron Microscopy Sciences) in Hanks’ Balanced Salt Solution (HBSS, Life Technologies, Carlsbad, CA, USA) for 30 min, followed by HBSS washing. Samples were permeabilized with Triton X-100 (0.1% *v*/*v*, 10 min, Sigma-Aldrich) in HBSS and were washed and incubated in blocking solution (1% *w*/*v* bovine serum albumin, BSA, Irving, TX, USA) for 1 h. Then, samples were incubated overnight at 4 °C with phalloidin/Alexa Fluor 488 (1:40, Molecular Probes-Invitrogen, Eugene, OR, USA) and rabbit anti-fibronectin antibody (1:400, F3648, Sigma-Aldrich). Afterward, samples were rinsed with HBSS and incubated for 45 min with Alexa Fluor 594 goat anti-rabbit secondary antibody (Molecular Probes-Invitrogen) and Hoechst for nuclei staining. Finally, samples were rinsed and confocal images were acquired using a laser scanning microscope (CLSM, Leica TCS-SP5 AOBS, Leica Microsystems, Wetzlar, Germany).

### 2.7. Immunohistochemistry Analysis

Skin equivalents were rinsed with HBSS, fixed in PFA for 30 min and extensively washed. Samples were then embedded in paraffin, sectioned onto 6 μm slides, and mounted on coverslips. Deparaffinized sections were subjected to heat-induced antigen retrieval using citrate buffer pH 6.0, permeabilized with 0.25% *v*/*v* Triton X-100, and rinsed with HBSS. Then, sections were blocked and incubated overnight with primary antibodies at 4 °C (Cytokeratin: 1:100, Dako; Vimentin: 1:100, Santa-Cruz Biotechnology, Dallas, TX, USA). Sections were rinsed and incubated with secondary antibodies (Cytokeratin: 1:1000, Alexa Fluor 488 donkey anti-rabbit; Vimentin: 1:1000, Alexa Fluor 488 donkey anti-mouse) for 1 h at room temperature, following extensive washing. After mounting, the sections were observed using a microscope (AxioImager Z1, Carl Zeiss, Jena, Germany).

### 2.8. Statistical Analysis

All statistical analyses were performed using GraphPad Prism 10. Data were presented as means ± SD and all statistical comparisons were made using a two-tailed Mann–Whitney test. The data presented are results from three independent experiments.

## 3. Results and Discussion

### 3.1. Rational Design of Dermal ECM-Inspired Bioinks

**Figure 1:**
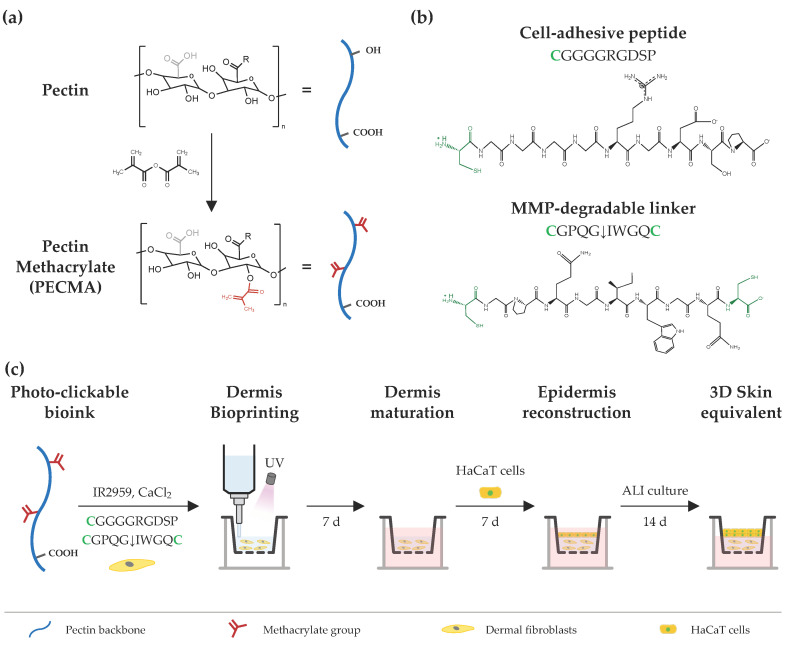
Schematic illustration of bioink design and extrusion bioprinting of 3D skin equivalents. (**a**) Chemical modification of pectin using methacrylic anhydride, yielding pectin methacrylate (PECMA), bearing methacrylates for photocrosslinking and carboxylic groups for binding with calcium. (**b**) Cell-adhesive and MMP-sensitive peptide sequences used for hydrogel biofunctionalization and photocrosslinking, respectively, via a thiol-ene reaction between methacrylates in the polymer backbone and cysteines (highlighted as green) in the peptide sequences. (**c**) A photocrosslinkable bioink is prepared from PECMA polymer and custom-made peptide sequences and its rheology is tuned via the addition of calcium chloride for ionic crosslinking, followed by mixing and homogenization to obtain a physically crosslinked bioink. Then, the bioink is loaded with dermal fibroblasts and bioprinted for dermal reconstruction, followed by in vitro dermis maturation. Afterwards, HaCaT cells are seeded onto the dermis, cultured under submerged conditions, and subsequently subjected to ALI culture to generate the bilayer skin equivalent.

The design strategy to engineer printable, ECM-mimetic bioinks comprises a single network polymer, complementary crosslinking mechanisms (ionic gelation and photocrosslinking), and custom peptide sequences (Figure 1). Pectin, a polysaccharide extracted from the cell walls of plants and fruits, was used as a representative bioink material due to its excellent biocompatibility, lack of cell responsive cues, and ease of crosslinking via ionic gelation [22]. The absence of cell-adhesive and protease-cleavage sites in the pectin backbone allows for fine tuning over cell–material interactions; for instance, via biofunctionalization with RGD-containing peptides or combination with cell-responsive biomaterials [23,24]. Furthermore, its rheological behavior can be controlled by exploring the native ability of pectin to form physically crosslinked hydrogels through the ionic interaction of carboxylic groups in the pectin backbone and divalent or trivalent ions in solution. Lastly, its mechanical properties can be modulated through either ionic crosslinking or the incorporation of functional groups (e.g., methacrylate) into the pectin backbone for photocrosslinking. Following this approach, the polymer was modified with methacrylates, yielding PECMA with a ~20% modification degree (Figure S1) [5], which undergoes photocrosslinking and retains the native ionic gelation ability. We took advantage of the ionic gelation of PECMA to obtain a printable bioink with structural stability during extrusion bioprinting through physical crosslinking of the polymer network using CaCl_2_. Ionic gelation proceeds in the presence of a water soluble photoinitiator (Irgacure 2959) and custom peptide sequences for 60 min to achieve complete gelation and obtain a pre-crosslinked material formulation with predictable rheology. Upon ionic gelation, human dermal fibroblasts were gently mixed with the pre-crosslinked PECMA, yielding a bioink that aids extrusion bioprinting. Then, the bioink was bioprinted onto a transwell and photocrosslinked for 40 s under UV light exposure, promoting the stabilization of the hydrogel network and imparting dermal-like mechanical properties through the formation of stable covalent bonds. After the maturation of bioprinted dermis for 7 days, human keratinocytes were manually seeded onto the dermis, cultured for 7 days under submerged conditions and, subsequently, exposed to ALI culture for 14 days.

Human dermis is composed of a dynamic ECM that provides embedded cells with cell-adhesion moieties to promote cell attachment and cell-ECM crosstalk, being susceptible to cell-mediated ECM remodeling via proteolytic degradation by cell-secreted matrix metalloproteinases (MMPs) [25]. As unmodified PECMA does not recapitulate such characteristics and prevents cell-material interactions, we selected two peptide sequences to promote cell adhesion (CGGGGRGDSP) and hydrogel network proteolytic remodeling (CGPQGIWGQC), recreating such properties of the dermis ECM. The peptide CGGGGRGDSP was selected as it contains the well-known fibronectin-derived RGD adhesion ligand sequence that imparts cell-adhesion to the polymer backbone and was used at a fixed concentration of 2 mM. The peptide crosslinker CGPQGIWGQC was used at a concentration of either 0.5 mM or 4 mM and was selected due to its susceptibility to cleavage by the MMPs secreted by skin cells [26,27]. The cell adhesive peptide was tethered into the polymer backbone via a light-activated thiol-ene click reaction between the methacrylates in the PECMA polymer and the thiol group from the cysteine amino acid in the peptide. The MMP-sensitive peptide was synthetized containing a cysteine amino acid in each peptide end, acting as a protease cleavable crosslinker that enables the formation of a covalently crosslinked hydrogel via a thiol-ene reaction. Notably, both peptides were tethered into the polymer backbone through a one-pot thiol-ene photo-click reaction, enabling simultaneous network biofunctionalization and hydrogel formation. This represents a major benefit of our design strategy as it precludes the need for multiple steps of peptide–polymer conjugation, purification, and peptide grafting quantification commonly used in polymer functionalization for bioink design.

Although we have selected two specific peptide sequences for bioink design, the versatility of our material system and thiol-ene reaction allows easy adaptation of the bioink composition to specific applications by incorporating cell-specific peptide sequences and/or crosslinkers. For example, it has been demonstrated that the functionalization of hydrogels with YIGSR peptide sequences promotes endothelial cell growth and organization in 3D hydrogels [28], while several bis-cysteine-containing peptide sequences have been engineered to exhibit degradation by specific MMPs secreted from different skin cells [29,30]. Furthermore, the use of bis-cysteine crosslinkers with different degradation rates has been demonstrated to be a straightforward strategy to tune the degradation of 3D hydrogels [31,32]. Using our design strategy, this knowledge can be translated to the rational design of bioinks containing cell-specific peptide sequences that can be employed, for example, to improve dermis vascularization or generate bioprinted skin substitutes with varying rates of degradation.

**Figure 2:**
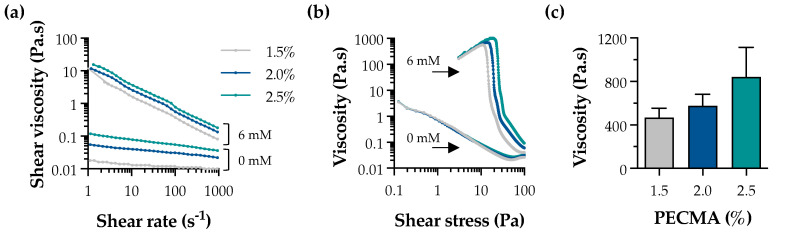
Rheological properties of pre-crosslinked PECMA inks. Effect of CaCl_2_ concentration (0 mM and 6 mM) on the shear viscosity (**a**) and yield stress (**b**) of inks prepared at varying polymer concentrations. (**c**) Viscosity of pre-crosslinked (6 mM CaCl_2_) PECMA inks at the yield point.

Ionic gelation is widely used for the formation of physical hydrogels exhibiting viscoelasticity [33]. In the context of extrusion bioprinting, ionic gelation can be used to assist the deposition of mechanically robust filaments via internal or external crosslinking. While internal gelation requires the use of an insoluble calcium salt (e.g., CaCO_3_ or CaSO_4_) and a pH decrease (e.g., D-glucono-δ-lactone) to promote the controlled exposure of cations throughout the polymer network, external gelation involves the diffusion of the multivalent cations from the outer region of the polymer, being characterized by a faster sol–gel transition [34,35]. External gelation has been applied to tune bioink rheology through pre-crosslinking, yielding a printable formulation, as well as for the bath bioprinting of ionically crosslinkable bioinks, promoting instantaneous filament crosslinking and stabilization [13,36]. Herein, external crosslinking was used to generate pre-crosslinked PECMA (bio)inks with controllable rheology and printability by crosslinking PECMA solutions prepared at varying polymer concentrations (1.5%, 2.0%, and 2.5%) using 6 mM CaCl_2_ (Figure S2). A concentration of 6 mM CaCl_2_ was selected based on our previous study, as it fits within the CaCl_2_ range that allows the formation of uniform pre-crosslinked inks, enabling the mixing of cells and aiding the extrusion bioprinting of 3D constructs of varying shapes and complexity with shape fidelity [5]. Shear rheology indicates that physically crosslinked inks exhibited a shear-thinning behavior (Figure 2a) and a clear yield stress (Figure 2b). The shear viscosity of pre-crosslinked inks was higher than those of non-crosslinked PECMA, while increased polymer content resulted in an enhanced viscosity due to the formation of more crosslinks in the gel network. Ionically crosslinked inks also exhibited a well-defined yield stress, which tends to increase with a higher PECMA content in the ink. Moreover, data from Figure 2b reveals that pre-crosslinked inks present a steep drop in viscosity with consequent material flow, which is characteristic of printable materials [37]. This is attributed to the increased density of carboxylic groups available for gelation, leading to a more crosslinked gel network. Indeed, this is corroborated by a trend in increased viscosity of the inks at the yield point (Figure 2c). As anticipated, non-crosslinked PECMA inks display a very low viscosity and do not present a clear yield stress.

### 3.2. Fabrication of Mechanically Tunable Dermal-like Hydrogels via Chain- and Step-Growth Mechanisms

Light-triggered thiol-ene click reactions are attracting increasing attention for bioink design due to their rapid reaction rate, efficiency under cell-compatible aqueous conditions, and spatial control over the gel network formation [13,38,39]. These reactions rely on the establishment of covalent linkages between thiol-containing compounds and alkenes in the presence of a photoinitiator, leading to the formation of chemically crosslinked hydrogels. In addition to UV light-mediated thiol-ene reactions, visible light photocrosslinking in the presence of ruthenium and sodium persulfate has also been explored to create pectin-based bioinks [40]. This reaction uses visible light for photocrosslinking and allows the crosslinking of methacryloyl or phenol groups, though longer crosslinking times are often required. Alternatively, enzymatic reactions using horseradish peroxidase and hydrogen peroxide have also been employed to create pectin hydrogels via a light-free reaction [41], but lacking spatiotemporal control over crosslinking.

**Figure 3:**
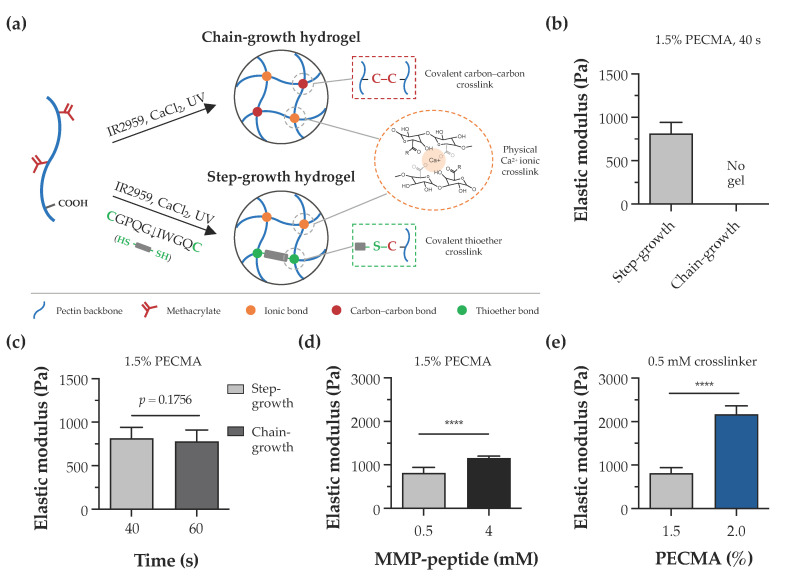
Hydrogel crosslinking and tunable mechanical properties. (**a**) Illustration of dual-crosslinked hydrogel network formed via either chain-growth or step-growth mechanisms, showing the establishment of ionic bonds via calcium crosslinking in both networks and the formation of chemical carbon–carbon crosslinks or thioether crosslinks, depending on the absence or presence of the peptide crosslinker, respectively. (**b**) Impact of chain-growth (no crosslinker) or step-growth (0.5 mM peptide crosslinker) mechanisms on the formation and mechanical properties of 1.5% PECMA hydrogels. (**c**) Mechanical properties of chain-growth and step-growth hydrogels (1.5% PECMA) prepared with varying photocrosslinking times. (**d**) Influence of peptide crosslinker content and (**e**) PECMA concentration on the mechanical properties of step-growth hydrogels (**** *p* < 0.0001).

In this study, the pectin backbone was modified with methacrylates to enhance the versatility of bioinks as they enable the photoinitiated hydrogel crosslinking via chain- or step-growth mechanisms. Despite other alkenes, such as norbornenes, exhibiting a superior reactivity to methacrylates, the limited to almost no norbornene homopolymerization requires a dithiol crosslinker for hydrogel formation [42]. As illustrated in Figure 3a, bioprinted calcium-crosslinked PECMA hydrogels can undergo a secondary crosslinking step via a free radical chain-growth mechanism by methacrylate homopolymerization. This is a straightforward reaction that proceeds in the presence of a photoinitiator and UV light. Alternatively, in the presence of a dithiol crosslinker (e.g., DTT, peptide), the crosslinking occurs via a step-growth mechanism involving a reaction between thiyl radicals and methacrylates with the formation of thioether linkages [43]. To assess the effect of crosslinking mechanisms in hydrogel formation, PECMA hydrogel precursors were prepared with or without 0.5 mM of biscysteine peptide crosslinker, followed by ionic crosslinking (6 mM CaCl_2_) and exposure to UV light for 40 s. Immediately after photocrosslinking, only PECMA hydrogels containing the peptide crosslinker underwent secondary crosslinking via a thiol-ene click reaction, enabling gel manipulation and remaining stable in culture medium. Mechanical characterization indicated that these hydrogels exhibit an elastic modulus of 814.0 ± 129.1 Pa after 24 h of incubation in culture medium (Figure 3b). In contrast, PECMA hydrogels lacking the peptide crosslinker did not establish enough density of covalent linkages after UV irradiation, resulting in a rapid loss of hydrogel structural integrity in the culture medium. However, mechanically stable PECMA hydrogels could be produced in the absence of the peptide crosslinker by increasing the UV exposure time to 60 s. This promotes the establishment of more covalent bonds in the gel network, contributing to increased mechanical integrity. Notably, the mechanical properties of chain-growth hydrogels crosslinked for 60 s (778.4 ± 132.0 Pa) were similar to those of step-growth hydrogels prepared with 40 s (814.0 ± 129.1 Pa) of UV irradiation (Figure 3c). These results suggest that, in the presence of the peptide crosslinker, secondary hydrogel crosslinking predominantly proceeds via a step-growth mechanism, owing to its faster reaction rate and efficiency. However, it is important to highlight that, under these conditions, hydrogels can be formed by a mixed mode polymerization in which methacrylate groups can react with either other methacrylates or thiyl radicals via a chain- or step-growth mechanism, respectively [44].

The dermis is the thickest skin layer that interfaces the epidermis and hypodermis and plays a fundamental role in mechanical integrity of the skin. The intricate composition of dermal ECM, made of collagen, glycoproteins, and proteoglycans, imparts the mechanical properties and regulates fundamental cell functions including spreading, migration, and proliferation [45,46]. Indeed, the mechanical properties of the cell microenvironment are recognized as a potent cue in directing cellular response and function. In the skin, several pathological conditions, such as cancer and fibrosis, are characterized by major alterations in the mechanical properties of ECM [47,48]. Despite several design strategies having been reported to create non-printable hydrogels with controllable mechanical properties [49,50], the design of material systems that recapitulate the mechanical properties of the dermis and provide a mechano-instructive microenvironment to embedded cells, while affording extrusion bioprinting is challenging. To evaluate the mechanical properties of hydrogels and demonstrate the mechanical tunability of our material system, double crosslinked PECMA hydrogels were prepared with varying concentrations of polymer and peptide crosslinker. The elastic modulus of 1.5% PECMA hydrogels increased from 814.0 ± 129.1 Pa to 1156.0 ± 49.13 Pa (a 1.42-fold increase) by enhancing the peptide crosslinker from 0.5 mM to 4 mM (Figure 3d). For a fixed content of peptide crosslinker (0.5 mM), a 2.66-fold increase in the elastic modulus was observed in hydrogels prepared using 2% PECMA content (2169.0 ± 194.8 Pa), compared to 1.5% (814.0 ± 129.1 Pa) (Figure 3e). Notably, the elastic modulus of printable, double crosslinked hydrogels falls within the range of human dermis, as determined using oscillatory rheometry [51]. Moreover, our material system enables us to control the mechanical properties of hydrogels through different routes, independently to the cell-adhesiveness of the gel network, providing the opportunity to present the cells with defined microenvironmental cues.

### 3.3. Bioprinted Dermal Equivalents with Tunable Mechanical Properties Modulate Site-Specific Responses of Dermal Fibroblasts in 3D

**Figure 4:**
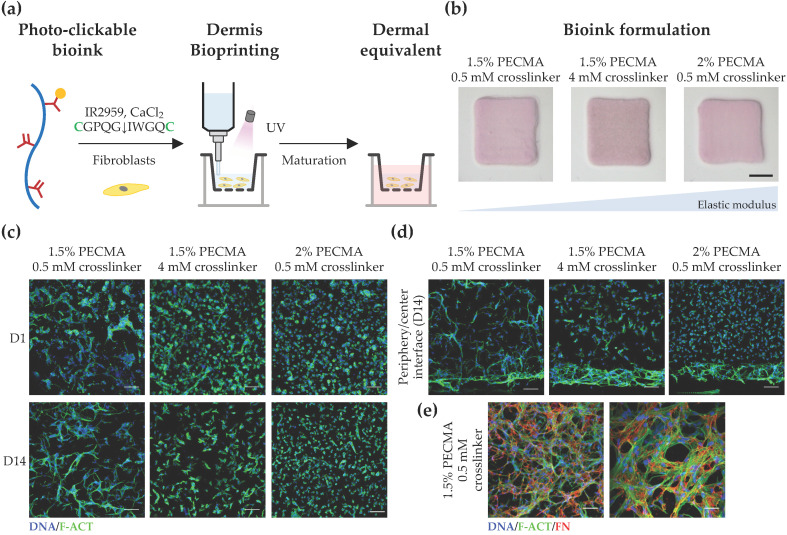
Bioprinting and characterization of dermal equivalents. (**a**) Bioprinting strategy to generate tissue-engineered dermis using dermal fibroblast-loaded thiol-ene bioink (cysteines highlighted as green). (**b**) Macroscopic images of bioprinted 3D dermal equivalents using bioinks with varying composition after 14 days of culture (scale bar: 2 mm). (**c**) Representative confocal images of fibroblasts stained for F-actin (F-ACT, green) and nuclei (DNA, blue), showing the effect of hydrogel elastic moduli on cell morphology at the center of the hydrogel (scale bar: 100 μm). (**d**) Cross-section confocal images of cells within bioprinted hydrogels stained for F-actin (green) and nuclei (blue) at day 14 (scale bar: 100 μm), showing the morphology of cells located at the hydrogel center and periphery. (**e**) Confocal images depicting the deposition of fibronectin (FN, red) within bioprinted dermis at day 14 (F-actin: green; nuclei: blue; left image scale bar: 100 μm; right image scale bar: 50 μm).

After demonstrating the mechanical tunability of the developed hydrogels, we next evaluated how mechanical properties impact the response of dermal fibroblasts in 3D-bioprinted dermal equivalents. To recapitulate the cell-adhesiveness and proteolytic remodeling of dermal ECM, fibroblast-laden PECMA (1.5% or 2.0%) bioinks were prepared using 6 mM CaCl_2_, 2 mM RGD peptide, and varying contents of MMP-sensitive crosslinker (0.5 mM or 4 mM), followed by bioprinting and culturing to promote dermis maturation (Figure 4a). Since a major limitation of collagen and decellularized ECM bioinks commonly used for skin bioprinting is the hydrogel contraction mediated by cell mechanical remodeling, which can limit the predictive value and hinder in vitro testing [52], the contraction of the bioprinted dermal constructs (6 × 6 mm^2^ and 1 mm thick) was evaluated at day 14 of culture. As can be seen in Figure 4b, despite the varying mechanical properties of bioprinted hydrogels (814.0 ± 129.1 Pa; 1156.0 ± 49.13 Pa; 2169.0 ± 194.8 Pa), there were no significant differences in hydrogel contraction. However, when we assessed the influence of environmental stiffness on cell spreading and morphology in 3D, significant differences were observed as a function of both culture time (Figure 4c) and spatial cell location (Figure 4d), though cells remained metabolically active in all constructs, regardless of the composition and crosslinking degree, indicating the cytocompatibility of bioinks (Figure S3). Specifically, fibroblasts embedded within 3D hydrogels displayed a stiffness-dependent cell morphology 24 h after bioprinting with cells cultured within low-stiffness hydrogels (814.0 ± 129.1 Pa), rapidly acquiring a spread and elongated morphology. Fibroblasts embedded within hydrogels of intermediate stiffness (1156.0 ± 49.13 Pa) exhibited a mixed cell morphology with some spread cells, while most of the cells cultured within stiff hydrogels (2169.0 ± 194.8 Pa) remained round. A similar behavior was observed after 14 days of culture with cells displaying a significant reduction in their ability to spread and establish cell–cell contacts with increased hydrogel stiffness. Despite hydrogels being crosslinked using the MMP-sensitive peptide that imparts embedded cells with the ability to degrade the gel network via the action of MMPs, similarly to what happens in the human dermis, our results suggest that the mechanical properties of the cell environment play a prominent role on cell morphology. However, detailed characterization regarding the secretion of MMPs by embedded cells throughout the culture time is required to elucidate the interplay between matrix mechanics and cell-mediated hydrogel degradation in the response of dermal fibroblasts. Our observations are corroborated by previous works, showing that fibroblasts are mechanosensitive and that their behavior in 3D hydrogels is highly dependent on the mechanical cues [53,54]. Moreover, in the context of skin models, it is important to highlight that presenting cells with non-physiological biomechanical cues can even lead to fibrosis onset and altered biological function via abnormal mechanosensing and mechanotransduction [55,56].

To further investigate the impact of hydrogel mechanical properties on cell response, we acquired cross-section confocal images of dermal fibroblasts and evaluated their morphology as a function of the spatial location in 3D (Figure 4d). Strikingly, a spread and elongated morphology was observed for cells located in the hydrogel periphery, regardless of the stiffness. Specifically, cells located in the periphery of hydrogels of intermediate and high stiffness formed a thicker layer of highly spread cells, a behavior which was not observed in low-stiffness hydrogels, where uniform cell spreading was detected throughout the gel. In fact, low-stiffness hydrogels promoted uniform cell colonization and the formation of multicellular networks in 3D. In contrast, heterogeneous and site-specific cell responses were detected in the remaining hydrogels, with round cells present in the center of stiffer gels and more elongated, yet isolated cells, being present in the center of gels with intermediate stiffness. We speculate that this behavior can be related to the mechanical confinement of dermal fibroblast cells, through which cells located in the hydrogel periphery encounter less resistance to mechanically probe and displace the matrix, while cells in the hydrogel center are more mechanically confined, driving distinct spatial cellular responses in 3D. Prior work has shown that the degree of spatial confinement is an important regulator of cell behavior (e.g., spreading, migration, and matrix deposition) and that cell fate is influenced by the interplay between the cell, degree of spatial confinement, matrix compliance, and degradability [57,58,59,60]. For example, high degrees of spatial confinement and matrix stiffness restrict cell migration into narrow confined spaces, due to the elevated energetic requirements for cell-induced matrix displacement during migration [58]. Furthermore, in viscoelastic alginate hydrogels, osteogenic differentiation of MSCs was inhibited when cell volume expansion is restricted by the mechanical properties of the matrix [59], while in non-degradable hyaluronic acid hydrogels, the extent of 3D cell confinement was coupled to matrix stiffness [60]. Based on these data, the bioink formulation (1.5% PECMA, 6 mM CaCl_2_, 2 mM RGD peptide, 0.5 mM MMP-sensitive crosslinker) that closely matches the dermis’ mechanical properties and promotes an in vivo-like dermal fibroblast response was selected to evaluate whether cells were able to secrete new ECM. As depicted in Figure 4e, an extensive network of fibrillar fibronectin, a classical protein of human dermis, was detected in bioprinted dermal analogues after 14 days of culture, indicating the ability of the developed bioinks and their tissue-like mechanical properties in supporting de novo ECM deposition.

### 3.4. Bioprinting 3D Bilayer Skin Models

**Figure 5:**
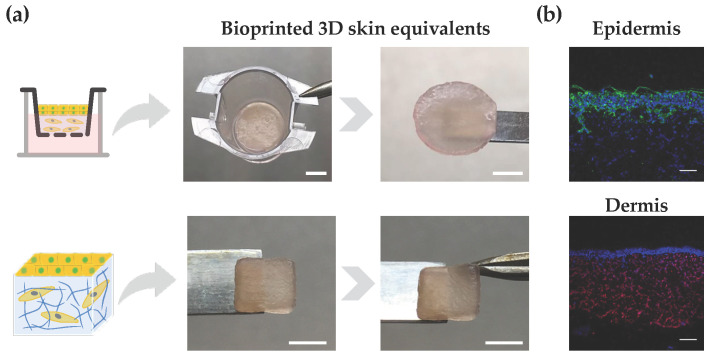
Bioprinted 3D skin models. (**a**) Illustration of bilayered models with circular and square shapes, as well as their structural integrity after 28 days of culture (scale bar top: 2.5 mm; scale bar down: 5 mm). (**b**) Immunostaining of paraffin-embedded samples using antibodies directed against cytokeratin (keratinocytes) in epidermis and vimentin (fibroblasts) in the dermis (epidermis: cytokeratin (green) and nuclei (blue); dermis: vimentin (red) and nuclei (blue); scale bar: 100 μm).

Lastly, we demonstrated the ability of the developed bioink in supporting the bioprinting and in vitro reconstruction of bilayer skin equivalents. As a proof-of-concept demonstration, the bioink loaded with dermal fibroblasts was extrusion bioprinted onto either a transwell system, which is widely used for drug screening and permeability studies, or a glass slide, resulting in 3D constructs with a user-defined shape (Figure 5a). After 7 days of dermis maturation, HaCaT cells were seeded on top of the dermis, cultured for an additional 7 days, and subjected to ALI culture for 14 days. Macroscopic images of 3D skin equivalents show their good mechanical integrity. While the transwell system imparted a characteristic circular shape to the constructs, we have also demonstrated the ability in bioprinting square-shaped constructs with good shape fidelity, which could be applied to generate personalized grafts for skin replacement. Moreover, it is also possible to observe the construct compliance to mechanical manipulation and the formation of a wrinkled surface resembling such behavior in native human skin. To assess the site-specific expression of key markers, immunohistochemistry analysis was performed on tissue sections obtained from the skin equivalents. The presence of cytokeratin positive cells was observed in the upper epidermal layer, while vimentin-stained cells were located in the lower dermal layer, confirming the site-specific localization of both cell types (Figure 5b). Some artifacts in the cytokeratin staining are attributed to sample processing and cutting, which can impact the integrity of the epidermis. Further research using the developed bioinks is warranted to evaluate the impact of the hydrogel’s mechanical properties on the fate of keratinocytes and the function of the reconstructed epidermis. Moreover, a fully automated bioprinting approach can also be implemented for the fabrication of multicellular bilayer skin models, by using either inkjet bioprinting or a spray valve to aid the automated dispensing on epidermal cells, including keratinocytes and melanocytes, on top of a mature dermis for epidermis reconstruction. It has been demonstrated that the automated deposition of epidermal cells contributes to improved skin pigmentation and epidermis formation, compared to manual cell seeding [61].

## 4. Conclusions

Novel ECM-inspired photo-clickable bioinks were designed for the bioprinting of skin models, whereby the bioink properties can be readily tuned to meet the rheological demands of extrusion bioprinting and the biological requirements of embedded cells. The bioinks display a unique set of properties, (i) enabling the fabrication of 3D constructs with a tissue relevant architecture, (ii) undergoing fast crosslinking without impairing cell viability, (iii) recapitulating the viscoelastic properties of the dermis, (iv) presenting the cells with relevant biochemical cues (adhesion motifs and degradation sites), and (v) stimulating tissue development. Using these bioinks, we found that mechanical properties are a powerful cue in directing the function of embedded dermal fibroblasts in 3D and spatially regulating their morphology and spreading. By mimicking the mechanical properties of the dermis, bioprinted constructs promoted similar cell behavior to the native tissue and enabled the reconstruction of bilayer skin models. More generally, these findings reveal the importance of providing tissue-mimetic mechanical cues to dermal fibroblasts towards generating biologically functional skin models and skin substitutes.

## Data Availability

Raw/processed data are available upon reasonable request addressed to the corresponding author.

## Supplementary Materials

The following supporting information can be downloaded at: https://www.mdpi.com/article/10.3390/biomimetics9040228/s1, Figure S1: ^1^H NMR spectra of pectin and pectin methacrylate (PECMA), indicating the appearance of two new peaks in PECMA (5.50–6.50 ppm) assigned to the methylene group in the vinyl bonds and a sharp peak at 1.90 ppm corresponding to the methyl group. Figure S2: Representative images (top: isometric view; bottom: top view) of bioprinted hollow constructs (∅ = 10 mm) created using ionically crosslinked PECMA inks (6 mM CaCl_2_). Inks were extrusion bioprinted into 10 layers constructs, sustaining the shape without photocrosslinking in a polymer concentration-dependent manner (scale bar: 5 mm). Figure S3: Metabolic activity of dermal fibroblasts within double crosslinked PECMA hydrogels (6 mM CaCl_2_, 2 mM RGD) prepared with varying polymer contents (1.5% and 2%) and MMP-peptide crosslinker concentrations (0.5 mM and 4 mM) at days 4 and 14 of culture (** *p* < 0.01, **** *p* < 0.0001).
