# Supplementary material for: Bioinspired and Photo-Clickable Thiol-Ene Bioinks for the Extrusion Bioprinting of Mechanically Tunable 3D Skin Models
Source: Biomimetics (Basel). 2024 Apr 10;9(4):228. doi: 10.3390/biomimetics9040228 (PMC11048463; doi:10.3390/biomimetics9040228)
Supplement: Supplementary file 1 [file biomimetics-09-00228-s001.zip › biomimetics-2911989-supplementary.pdf]

biomimetics

MDPI

Supporting Information

Bioinspired and Photo-Clickable Thiol-Ene Bioinks for the Extrusion Bioprinting of Mechanically Tunable 3D Skin Models

Luís B. Bebiano [1,2], Rafaela Presa [1,2,3], Francisca Vieira [1,2], Bianca N. Lourenço [1,2] and Rúben F. Pereira [1,2,3,*]

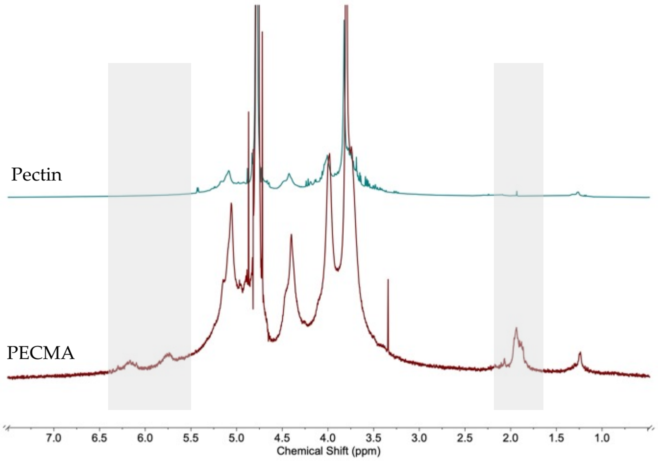

Figure S1. ^{1}H NMR spectra of pectin and pectin methacrylate (PECMA), showing two new peaks in PECMA (5.50-6.50 ppm) assigned to the methylene group in the vinyl bonds and a sharp peak at 1.90 ppm corresponding to the methyl group. Degree of methacrylation is ~20% and was calculated as detailed [5].

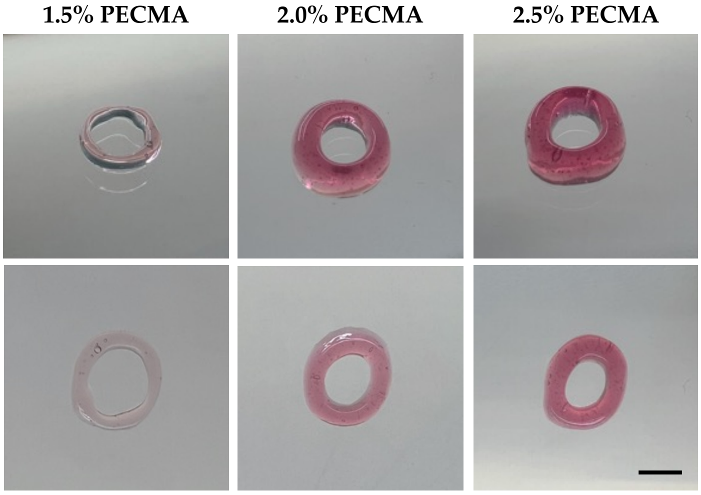

Figure S2. Representative images (top: isometric view; bottom: top view) of bioprinted hollow constructs (∅ = 10 mm) created using ionically crosslinked PECMA inks (6 mM $CaCl_2$). Inks were extrusion bioprinted into 10 layers constructs, sustaining the shape without photocrosslinking in a polymer concentration-dependent manner (scale bar: 5 mm).

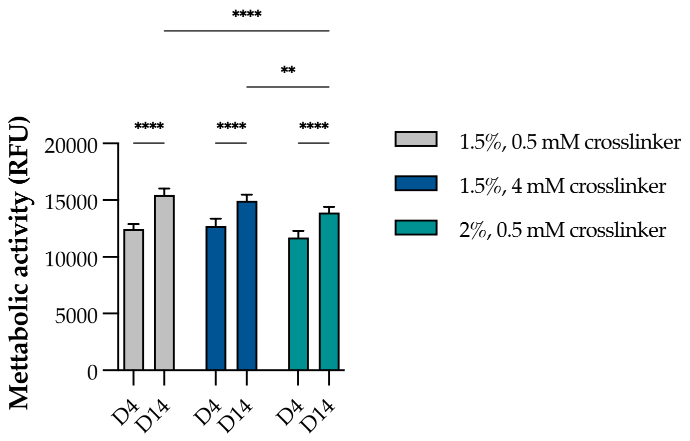

Figure S3. Metabolic activity of dermal fibroblasts within double crosslinked PECMA hydrogels (6 mM $CaCl_2$, 2 mM RGD) prepared with varying polymer contents (1.5% and 2%) and MMP-peptide crosslinker concentrations (0.5 mM and 4 mM) at days 4 and 14 of culture (**$p < 0.01$, ****$p < 0.0001$).
